# Persistence of Multi-Drug Resistance Plasmids in Sterile Water under Very Low Concentrations of Tetracycline

**DOI:** 10.1264/jsme2.ME15122

**Published:** 2015-12-04

**Authors:** Thi Lan Thanh Bien, Yuki Sato-Takabe, Mitsuko Ogo, Masaru Usui, Satoru Suzuki

**Affiliations:** 1 Center for Marine Environmental Studies (CMES), Ehime UniversityBunkyo 3, Matsuyama, Ehime 790–8577Japan; 2 United Graduate School of Agricultural Science, Ehime UniversityTarumi 3, Matsuyama, Ehime 790–8566Japan; 3 Department of Biotechnology, Nong Lam UniversityLinh Trung Ward, Thu Duc District, Ho Chi Minh CityVietnam; 4 Rakuno Gakuen University School of Veterinary MedicineEbetsu, Hokkaido 069–8501Japan

**Keywords:** multi-drug resistance plasmids, persistence, non-culturable, starvation, reservoir

## Abstract

The persistence of the multi-drug resistance plasmids pAQU1 and IncFIB was examined in bacterial populations under very low selective pressure. We herein demonstrated that these plasmids stably remained not only in the original host, but also in a transconjugant, even after being in a non-culturable state. In seawater microcosms containing *Photobacterium damselae* 04Ya311 possessing pAQU1, no significant loss of pAQU1 was observed during a 30-d starvation period. The copy numbers of pAQU1 and IncFIB in *E. coli* were constant. The results of the present study suggest that these plasmids have the ability to remain among various bacteria under oligotrophic conditions with low antibiotic selection pressure.

Plasmids are transferred into and replicate in various bacterial hosts (9), thereby allowing bacteria to grow in the presence of antibiotics if antibiotic-resistance genes (ARGs) are conveyed (11, 14). The management and control of ARGs are important for preventing damage by antibiotic-resistant pathogens. Plasmid stability in bacterial populations, particularly when selective pressure is very low or absent, is fundamental as a countermeasure against antibiotic resistance. The theory of plasmid persistence in experimental conditions has already been described (6, 9, 36); however, most of this theory was obtained under experimental conditions using cultured bacteria. Plasmid dynamics in a marine environment currently remain unclear. The natural persistence of plasmids in water environments under oligotrophic and non-selective conditions needs to be examined in order to evaluate the reservoir bacteria of ARGs.

Plasmid-free (ARG-free) cells are eliminated, whereas plasmid-containing cells thrive in the presence of antimicrobial agents, resulting in an increasing number of resistant cells containing plasmids in bacterial populations (43). The stability of ARGs and plasmids is generally related to the fitness cost for hosts (5, 25). The elimination of ARGs and plasmids may reduce the fitness burden of bacteria without selective pressure (3). Sode *et al.* (35) previously reported that 50% of pKT230 plasmids harbored by *Synechococcus* sp. were lost without antimicrobial pressure. This finding suggests that the prevalence of established antibiotic resistance decreases after reducing the use of antibiotics (19). However, the maintenance and spread of ARGs conveyed to plasmids among bacterial populations in a natural environment without antimicrobial selective pressure have not yet been examined in detail. Since some resistance mechanisms may be costfree, bacteria may have the ability to overcome the fitness costs of resistance by compensatory evolution, which restores fitness without the loss of resistance (24).

*Photobacterium damselae* subsp. *damselae*, a member of the family Vibrionaceae, is a marine bacterium that causes opportunistic infections in marine animals and humans (31). Strain 04Ya311 of *P. damselae* subsp. *damselae*, which was isolated from seawater in a coastal aquaculture site in Japan, harbors the novel multi-drug resistance plasmid pAQU1 (26). “pAQU series” plasmids have been detected in other marine species (27) and are involved in the conjugative transfer of ARGs (26, 27). pAQU1 relates to the incompatible plasmid IncA/C in terms of transposition genes (*tra* series) (26). Approximately 30 incompatible plasmids have been identified to date (7). These plasmids cannot simultaneously co-exist in one cell because of the interference of replication (28). IncA/C-type plasmids have recently become a major environmental concern (29) because they have a broad host range and are capable of spreading multi-drug resistance via conjugative transfer within bacterial communities (20). However, the mechanisms used by these plasmids to reside in aquatic environments have not yet been determined despite their frequent detection.

Previous studies reported that conjugative plasmids cause little or no fitness burden on bacterial hosts (6, 12). In contrast, Subbiah *et al.* (36) demonstrated that the carriage of *bla*_CMY-2_-positive IncA/C plasmids imposed a measurable fitness cost on a host, leading to the decay and loss of plasmids during a long-term passage without antibiotic selection. Furthermore, one plasmid may be stable in one strain, but unstable in others (9). Since antibiotic-resistant bacteria and ARGs have been widely detected, even in non-contaminated environments (33), ARG risk assessments are needed. ARGs present in various environments may be a natural phenomenon (8, 22), the result of transmission from humans, or both (23).

Multi-drug resistance conjugative plasmids including the IncA/C type are widely distributed among marine and enteric bacteria (20, 26). When enteric and pathogenic bacteria are released into natural environments (17), they often enter a viable but non-culturable (VNC) state in response to adverse environmental conditions (30). Furthermore, most aquatic bacteria are non-culturable or yet-to-be cultured bacteria (2, 4, 40). Consequently, it has yet to be established whether plasmids are retained in and transferred to and from natural culturable and non-culturable bacteria. A sulfamethoxazole resistance gene (*sul*3) was previously reported to be specifically distributed in a non-culturable seawater assemblage, but not in a culturable assemblage. The *sul1*, *sul2*, and *sul3* genes were found to be present at an almost undetectable level in a freshwater assemblage (39). Based on these findings, we hypothesize that plasmids with various origins stably reside among a natural assemblage, in which the majority in seawater have the ability to form a large reservoir in the natural environment.

Therefore, the aim of the present study is to determine the persistence statuses of plasmids in different host strains in water, which is important for an appropriate assessment of whether environmental ARGs pose a risk. We prepared defined microcosms and examined the persistence of the multi-drug resistance plasmid, pAQU1, in the host *P. damselae* subsp. *damselae* 04Ya311 and transconjugant *E. coli* W3110 under starvation conditions with very low concentrations of tetracycline. In order to investigate the effects of this plasmid on bacterial host fitness in more detail, the transferable incompatibility FIB group plasmid, IncFIB, in *E. coli* 133 isolated from cattle feces (42) was used as a comparison.

*Photobacterium damselae* subsp. *damselae* 04Ya311 and *E. coli* W3110 cells harboring pAQU1 were used in the present study. The pAQU1 (204 kb) plasmid contained seven ARGs: *bla*_CARB-9_-like, *flo*R, *mph*(A)-like, *mef*(A)-like, *sul2*, *tet*(M), and *tet*(B) (26). Among its various multi-drug resistance properties, we used tetracycline resistance as the selective marker for pAQU1. The minimal inhibitory concentrations (MIC) of tetracycline (TC; Nacalai Tesque, Kyoto, Japan) were 64 μg mL^−1^ (the 04Ya311 strain) and 128 μg mL^−1^ (the W3110-transconjugant strain), as determined by E-test (BioMérieux, USA). These strains were pre-cultured in 10 mL Mueller-Hinton Broth (MHB; Becton, Dickinson and Company, Franklin Lakes, USA) with 2% NaCl and Luria Bertani medium (LB; BD), respectively. TC (20 μg mL^−1^) was supplemented in pre-culture media. Overnight cultures were harvested by centrifugation (8,000×*g*, 10 min), and cells were then washed three times and resuspended in 10 mL sterile natural seawater for 04Ya311 and sterile well water for W3110. Some cells were added to 100 mL of 0.45-μm filtered and sterilized natural seawater collected at Uwa Sea, south Ehime, Japan (pH 8.3, salinity 30.3) or natural well water in Ehime University (pH 7.2, salinity 0) with TC at concentrations of 0, 0.01, 0.1, 1.0, and 10 μg mL^−1^. Liquid cultures were incubated at 25°C for 04Ya311 and 37°C for W3110 for up to 30 d in a shaking incubator (100 rpm). Subsamples (5 mL) were withdrawn at various times for cell counting and DNA extraction. As a comparison, the plasmid IncFIB (120 kb) carrying the *bla*_CTX-M15_, *bla*_TEM_, and *tet*(A) genes in *E. coli* strain 133 (42) was used.

The total number of bacterial cells was directly enumerated using a 4′,6-diamidino-2-phenylindole (DAPI) staining method as previously described (32) with a slight modification in that cells were collected on a 0.22-μm black polycarbonate membrane filter (Advantec, Dublin, CA, USA), and counted with an Olympus BX51 fluorescence microscope (Olympus Optical, Tokyo, Japan). TC-resistant bacteria were counted on TC-containing media that selected plasmid-containing cells. Samples diluted 10-fold were plated on MHB with 2% NaCl or LB containing 1.5% Bacto agar (BD) and 20 μg mL^−1^ TC. The plates were then incubated for 24 h, and the number of colony-forming units (CFU) was counted.

Regarding total DNA extraction, 1 mL of the culture solution was filtered through 47-mm polycarbonate filters with a pore size of 0.22 μm (Millipore, USA), and stored at −20°C until use. Total DNA was extracted from the filters according to the method described by Dempster *et al.* (10).

In order to quantify plasmid copy numbers, the TC resistance genes *tet*(M) and *tet*(A) were targeted for pAQU1 and IncFIB, respectively. There was one copy of these *tet* genes on each plasmid. PCR primer sets have previously been reported for *tet*(M) (41) and *tet*(A) (21). Amplicon sizes were 186-bp (*tet*[M]) and 387-bp (*tet*[A]). The copy number of the *tet* genes was normalized using the 16S rRNA gene copy number (38). Quantitative real-time PCR (qPCR) was performed using the CFX96 Real-time System (Bio-Rad Laboratories, Hercules, CA, USA). The qPCR program for 16S rRNA genes was described previously (39), and that for *tet*(M) and *tet*(A) was at 95°C for 30 s, 40 cycles at 95°C for 10 s for *tet*(M) and 15 s for *tet*(A), and then at 57°C for 20 s for *tet*(M) and 55°C for *tet*(A). The following plasmids were used as standard curves: the pGEM-T Easy vector harboring the 16S rRNA gene from *E. coli* K12 (39), pGEM-tetM (22), and pGEM-T carrying a 1.367-kb PCR product with the *tet*(A) gene amplified with a primer set (forward-5′-GCACG GATCACTGTATTCG-3′, and reverse-5′-CATGGCATAG GCCTATCG-3′) from the plasmid IncFIB.

The results of time-course changes in bacteria and plasmids are shown in Fig. 1. In the original host *P. damselae* subsp. *damselae* 04Ya311 (Fig. 1A a, b, and c), the initial numbers of total bacterial cells (Fig. 1a) and plasmid-containing cells (Fig. 1b) were 3.4×10^7^ cells mL^−1^ and 1.4×10^6^ CFU mL^−1^, respectively. The copy number of pAQU1 in ratio to the 16S rRNA gene copy number (16S) was 6×10^−1^ copies (Fig. 1c). The numbers of total cells and plasmid-containing cells at all TC concentrations were approximately 10-fold higher 1 d after the start of the incubation and then remained stable with no significant reductions until after 10 d. This result indicated that none of the TC concentrations used significantly affected the growth of 04Ya311 within 10 d of starvation, and this may enhance host fitness by increasing the bacterial growth rate without selective pressure (12). Our results are consistent with previous findings (37) in which low concentrations of oxytetracycline (OTC) in the water phase did not change bacterial community structures; however, low levels of antibiotics may allow for the selection and enrichment of resistant bacteria (16). Another possibility is the induction of horizontal gene transfer (18). The number of TC-resistant cells (plasmid-containing cells) slightly decreased to 3×10^4^ CFU mL^−1^ (10 μg mL^−1^ TC addition) after 17 d and to 1.9–4.4×10^5^ CFU mL^−1^ (0–1.0 μg mL^−1^ TC addition) after 30 d (Fig. 1b). The CFU number for a high TC concentration (10 μg mL^−1^) was approximately 10-fold lower than that for low TC concentrations (0–1.0 μg mL^−1^). Although the copy number of pAQU1 in the 04Ya311 population varied, it remained at approximately 10^−1^ copies per 16S during the incubation period (Fig. 1c). These results indicate that pAQU1 stably persists in an original host population at all concentrations of TC. Regarding the marked fluctuations observed in the plasmid copy number shown in Fig. 1c, we speculate that none of the 04Ya311 cells possessed uniform numbers of the plasmid, whereas the plasmid possession status of the transconjugant was uniform. The MIC of 04Ya311 for tetracycline was 64 μg mL^−1^, whereas that of W3110 was 128 μg mL^−1^. This variation in plasmid numbers may cause a lower MIC in the 04Ya311 community.

In transconjugant *E. coli* W3110 having pAQU1 (Fig. 1B d, e, and f), the total number of bacterial cells (Fig. 1d) was stable at approximately 10^7^ cells mL^−1^ throughout the study period, whereas the number of plasmid-containing cells (Fig. 1e) gradually decreased to less than the detection limit after 25 d. This decrease may have been due to bacteria being injured, stressed, or entering a non-culturable state (1, 30). However, the copy number of pAQU1 was stable at approximately 10^−1^ copies per 16S until 30 d (Fig. 1f), which suggests that pAQU1 has the ability to stably reside in nonculturable cells. Another profile was observed for IncFIB in *E. coli* 133 (Fig. 1C g, h, and i), with the copy number of IncFIB being stable at approximately 10^1^ copies per 16S (Fig. 1i); however, the number of plasmid-containing cells decreased slightly (Fig. 1h). Plasmid copies/16S did not decrease in either *E. coli* strain, suggesting that the *E. coli* population is a reservoir even after being in a non-culturable state in an environment without selection pressure; however, each strain displayed different responses in well water.

In order to examine the effects of plasmids on host fitness, we measured time-course changes in cell numbers for the plasmid-free strain *E. coli* W3110 and plasmid-cured strain *E. coli* 133 in sterile well water (Fig. 2). The plasmid curing of strain 133 was performed as described previously (34). In this experiment, we used a flavophospholipol (64 mg L^−1^) as a plasmid-curing agent (13). Both strains showed similar profiles (Fig. 2) in that CFU numbers gradually decreased, whereas the total cell number did not change during the incubation. A decrease in the CFU of W3110 was observed under conditions with and without the plasmid (Fig. 1e and 2a), while plasmid-cured 133 entered a non-culturable state more rapidly than with IncFIB (Fig. 1h and 2b). This result suggests that plasmids impose no cost on or improve the fitness of their hosts. These results are consistent with the findings of Enne *et al.* (12) in which the *sul2*-coding plasmid p9123 conferred a 4% fitness advantage on its original clinical host in the absence of selective pressure. Dionisio *et al.* (11) showed that evolution of the R1 conjugative plasmid (a member of the IncFII group) in *E. coli* markedly increased the fitness of other bacterial cells, including *Salmonella*. The results of the present study imply that various hosts play the role of reservoirs in freshwater and seawater environments. Our results are inconsistent with those of Subbiah *et al.* (36) who demonstrated that selective pressure was required for the long-term persistence of *bla*_CMY-2_-positive IncA/C plasmids in *E. coli* and *Salmonella*. On the other hand, Cottell *et al.* (6) reported that the transferable extended-spectrum-β-lactamase-carrying plasmid persisted and spread without antimicrobial selective pressure. Consequently, plasmids that persist and have a strong conjugated system have the advantage of overcoming the evolution of the bacterial host (15). These differences may be caused by *par*A and *par*B (20, 28) in plasmids, which function to ensure the proper partition of the plasmid into daughter cells (28), and pAQU1 has this partitioning machinery (26), which may cause an accurate partition without selection pressure.

To date, culture-dependent methods have been commonly used to detect antibiotic resistance because they are needed in order to identify the phenotype of “resistance”. However, one limitation of this method is the underestimation of the number of plasmid-containing bacteria that are injured, stressed, or in a VNC state (1). The results presented in Fig. 1 suggest that qPCR combined with a culture-dependent method and DAPI counting is useful for determining plasmid dynamics in a microbial community containing cells under various conditions.

In conclusion, multi-drug resistance plasmids may be stably retained not only in the original host, but also in a transconjugant without selection pressure even after being in a non-culturable state, despite plasmids being considered nonessential for bacteria survival. Our results suggest that the elimination of a plasmid from a natural environment is unlikely.

## Figures and Tables

**Fig. 1:**
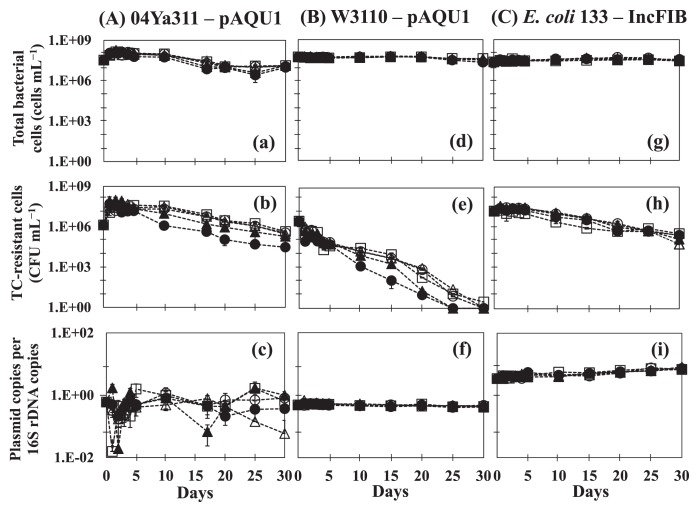
Profiles of pAQU1 in *P. damselae* subsp. *damselae* 04Ya311 (A, a–c) and in transconjugant *E. coli* W3110 (B, d–f), and IncFIB in *E. coli* 133 (C, g–i) at TC concentrations of (○) 0 μg mL^−1^, (□) 0.01 μg mL^−1^, (△) 0.1 μg mL^−1^, (▲) 1.0 μg mL^−1^, and (●) 10 μg mL^−1^. The total bacterial cell number (a, d, g), the colony-forming bacterial number on medium selected for plasmid-containing cells (b, e, h), and the plasmid copy number per 16S rRNA gene (c, f, i) are shown. Error bars were obtained from triplicate measurements.

**Fig. 2:**
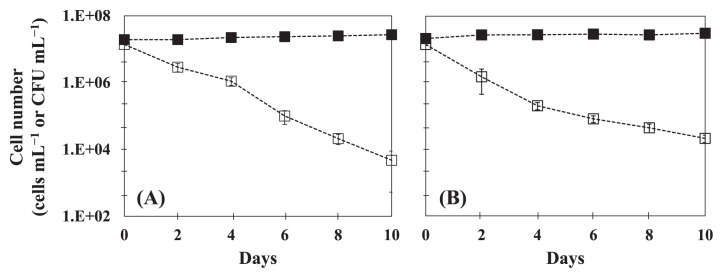
Time-course changes in cell numbers of plasmid-free *E. coli* W3110 (A) and plasmid-cured *E. coli* 133 (B). Symbols are: (■) total cell number (cell mL^−1^) by DAPI counting and (□) colony-forming number (CFU mL^−1^). Error bars were obtained from two independent experiments.
